# Comparison of performance of Sri Lankan and US children on cognitive and motor scales of the Bayley scales of infant development

**DOI:** 10.1186/1756-0500-7-300

**Published:** 2014-05-16

**Authors:** Pavithra Godamunne, Chathurika Liyanage, Nalin Wimaladharmasooriya, Arunasalem Pathmeswaran, Ananda Rajitha Wickremasinghe, Cryshelle Patterson, Nalini Sathiakumar

**Affiliations:** 1 Faculty of Medicine, University of Kelaniya, Ragama, Sri Lanka; 2 Civitan-Sparks Clinics, University of Alabama, Birmingham, USA; 3 School of Public Health, University of Alabama, Birmingham, USA

**Keywords:** Bayley III scales, Cognitive, Motor, Sri Lanka, Children 6–24 months

## Abstract

**Background:**

There is no validated scale to assess neurodevelopment of infants and children in Sri Lanka. The Bayley III scales have used widely globally but it has not been validated for Sri Lankan children. We administered the Cognitive and Motor Scales of the Bayley III to 150 full-term children aged 6, 12 and 24 months from the Gampaha District of Sri Lanka. We compared the performance of Sri Lankan children 6, 12 and 24 months of age on the cognitive and motor scales of the Bayley III with that of US children.

**Results:**

Compared to the US norms, at 12 months, Sri Lankan children had significantly higher cognitive scores and lower gross motor scores, and at 24 months significantly lower cognitive scores. The test had a high test-retest reliability among Sri Lankan children.

**Conclusions:**

There were small differences in the cognitive and motors scores between Sri Lankan and US children. It is feasible to use Bayley III scales to assess neurodevelopment of Sri Lankan children. However, we recommend that the tool be validated using a larger representative sample of all population groups.

## Background

Early intervention in children at risk for developmental delays can lead to better outcomes and improved child well-being. The prevalence of developmental disabilities in Sri Lanka has been estimated to be 12 – 29% and the majority of children with clinically identifiable developmental problems in Sri Lanka are referred to child mental health services relatively late, generally when they are over 5 years of age, due to low rates of early recognition [1]. Currently, a culturally validated Denver Development Screening Test (DDST) is being used in the Maternal and Child Health programme of the Ministry of Health, Sri Lanka to screen child development. The Denver II has been shown to have limited specificity and high over-referral rates [2] and has been considered as test of questionable value in terms of screening for developmental delay [3]. Furthermore, it is only a screening tool and is not useful for diagnostic purposes. A reliable and valid tool for diagnosing developmental delays in very young children would enable the early identification of children with impairments. The Bayley Scales of infant development is a commonly used psychometric tool for assessing the development of children between 1 to 42 months of age. It has been shown to be a valid diagnostic tool for identifying children with developmental delays at an early age and is widely used in clinical settings due to its solid theoretical background and robust psychometric properties [4]. The Bayley scales have been used in various countries such as Brazil [5], Taiwan [4] and Australia [6]; currently it is in its third edition [7]. The Bayley III scales can be used to assess infant and toddler development across five key developmental domains - cognition, language, social-emotional, motor and adaptive behaviour [8]. The interpretation of Bayley scores are based on norms established for children in the United States (US), which is based on a national standardized sample of 1700 children between 1 and 42 months of age, and divided into 17 age groups.

Despite its wide use and well-established psychometric properties, the Bayley III scale has not been validated in Sri Lanka. Socio-cultural influences can occur early in life and affect cognitive, sensorimotor and socio-emotional domains of behaviour [9] which may result in differences in the cross cultural application of the Bayley Scales for the assessment of infants. For example, Taiwanese children had lower scores on the Bayley cognitive and motor scales when compared to the US norms at six and 24 months [4] and Brazilian children aged between one and 12 months had lower motor scores [5]. These differences may be due to heredity, differences in socio-economic status and child-rearing practices [4]. Hence, the use of US norms to assess the development of children from other cultures may not be appropriate; it is important to establish normative data for Sri Lanka children for the instrument to be used as a diagnostic tool in the clinical setting.

This manuscript compares the performance of Sri Lankan children 6, 12 and 24 months of age on the cognitive and motor scales of the Bayley III with that of US children.

## Methods

### Participants

Infants and toddlers aged 6, 12 and 24 months (+2 weeks) residing in the Ragama and Wattala Medical Officer of Health (MOH) areas in the Gampaha District of Sri Lanka participated in the study. Infants who were registered with the Public Health Midwife (PHM) of the area, and visiting the community child welfare clinics were recruited for the study. A total of 150 Sri Lankan children participated in the study, with 50 children from each age group. Comparison of 50 Sri Lankan children (SD = 2.0) with 100 US children (SD = 3.0) to detect a difference in means of 1.0 assuming a two-sided alpha error of 0.05 has a power of 68%. Only full-term infants were considered for enrollment and pre-term infants were excluded. Prematurity was defined as a “gestation period of ≤36 weeks” (Bayley, [7], p28). As the objective of the study was to identify mean scores for apparently normal children, children with a birth weight of <2500 g and children with diagnosed acute, chronic or congenital medical conditions such as progressive neurological disorders, congenital heart disease etc., or children with developmental delays based on the medical history and the entries made in the Child Health Development Record (CHDR) were excluded. Children of mothers who experienced complications such as hypertension or gestational diabetes during the pregnancy of the child were also excluded.

### Research design

A cross sectional study design was used.

### Test administration procedure

Motor behavior is considered to be one of the best indicators of overall well-being in infants in the first year of life [5] and cognitive scales provide an indication as to whether a young child has achieved a typical developmental level. Hence, the cognitive scale and the motor scale of the Bayley III were used to get an overall picture of Sri Lankan children’s development in this study. The cognitive scale and the fine motor and gross motor sub tests of the motor scale of Bayley III were administered to selected children by a psychologist and two medical graduates. The test administrators were rigorously trained prior to conducting the study as recommended in the guidelines of Bayley III [7]. The psychologist was trained by a licensed clinical psychologist at the University of Alabama at Birmingham, USA; the two medical graduates were trained in Sri Lanka by the psychologist. To ensure the maintenance of accurate test administration and scoring, the following procedures were employed: the initial 20 administrations of the Bayley scales were co-scored by the two testers and 20% of all test protocols were re-scored for accuracy by the test administration supervisor i.e. the psychologist. Any tester making more than one mistake on a protocol had their following three protocols re-scored for accuracy. One in every four test administrations were directly observed by the test administration supervisor, who co-scored a protocol as the test was administered.

The instructions to children for the cognitive and motor scales were translated into Sinhala, the local language, by the principal investigator and back-translated to English by an independent person. The two English versions were found to be comparable. Participant children were all administered the test at a designated testing centre ensuring uniform test administration procedures being maintained throughout the study.

The cognitive scale, the fine motor and gross motor subtests of Bayley III were administered on participating children according to the guidelines of the manual [8]. Each administration took between 20 and 90 minutes, depending on the age of the child; the administration of the scale on older children required more time.

The three scales were re-administered on 10% of the total sample (n = 15) to establish test-retest reliability of the scales. Two testers simultaneously scored the tests for the test-retest sample on both occasions of the test administration. The test-retest reliability was assessed by the intra-class correlation (ICC) coefficient based on a one-way random effects ANOVA model to test for absolute agreement in the ratings using SPSS.

### Comparison of scores

Raw scores were calculated for each sub test by adding up the scores obtained for a subtest; scaled scores denote a testee’s performance on a particular subtest compared to his/her same aged peers. The raw scores calculated for the Bayley cognitive, fine motor and gross motor scales for each participant were converted into scaled scores using the tables in the Bayley manual. The scaled scores, derived from the total raw scores on each of the subtests, have been scaled to a metric with a range of 1 to 19, a mean of 10, and a standard deviation of 3. For any age group, the average performance on a subtest would be a scaled score of 10 [8]. The mean scaled scores and standard deviations for the 3 subscales for each age group were compared with the US norms using t-tests.

### Ethical considerations

Ethics approval was obtained from the Ethics Committee of the Faculty of Medicine, University of Kelaniya, Sri Lanka. All participants were volunteers. Written informed consent was obtained from a parent prior to administration of the test to children.

## Results

The socio-demographic information of the participant children are presented in Table 1. 57% (n = 85) of the participant children were males (Table 2). 60% (n = 90) of the children had at least one sibling, and of those children having a sibling, 97.8% (n = 88) had an older sibling(s).

**Table 1:** Socio-economic characteristics of participant children

**Characteristic**	**Number**	**Percentage**
**Ethnicity**		
Sinhalese	128	85.3
Tamil	12	8.0
Moor	4	2.7
Burgher	4	2.7
Other	2	1.3
**Religion**		
Buddhist	96	64.0
Catholic/Christian	40	26.7
Hindu	9	6.0
Islam	5	3.3
**Monthly income (SLR)**^**1**^		
< 5,000	4	2.7
5,001 – 15, 000	48	32.0
15, 001 – 25,000	59	39.3
25,001 – 50, 000	29	19.3
> 50, 000	10	6.7
**Mother’s educational level**		
Less than G.C.E. O/Ls	25	16.7
Upto G.C.E. O/Ls	72	48.0
Upto G.C.E A/Ls	46	30.7
Graduate and above	4	2.7
Other professional/technical	3	2.0
**Father’s educational level**		
Less than G.C.E. O/Ls	25	16.7
Upto G.C.E. O/Ls	73	48.7
Upto G.C.E A/Ls	47	31.3
Graduate and above	4	2.7
Other professional/technical	1	0.7
**Total number of children**	150	100

^1^1 USD = SLR 130.00.

**Table 2:** Distribution of children by age and gender

**Age group**	**Number of males**	**Number of females**
6 months	22	28
12 months	29	21
24 months	34	16
**Total**	**85**	**65**

There were no differences in the cognitive, fine motor and gross motor scales of Bayley III between 6 month old Sri Lankan (SL) and US children (Table 3). Sri Lankan children scored significantly higher on the cognitive scale (p = 0.001) at 12 months and significantly lower (p = 0.048) at 24 months as compared to US children. At 12 months, Sri Lankan children scored significantly less on the gross motor subtest as compared to US children (p = 0.034); at 24 months, there was no difference in the gross motor subtest scores between the two groups of children. There were no differences in the fine motor subtest scores between the two groups of children at any of the ages considered.

**Table 3:** Comparison of performance of Sri Lankan and US children on the cognitive and motor scales of the Bayley scales of infant development

**Age group**	**Subtest**	**Sri Lankan children (n = 50)**^**1**^		**US children (n = 100)**		**Mean difference**	**95% confidence interval of the difference**		**p-value**
		**Mean**	**SD**^**1**^	**Mean**	**SD**^**2**^		**Lower**	**Upper**	
6 months	Cognitive	9.860	1.730	10.000	3.000	-0.1400	-1.046	0.766	0.761
	FM^3^	10.640	1.940	10.000	3.000	0.6400	-0.283	1.563	0.171
	GM^4^	10.480	2.050	10.000	3.000	0.4800	-0.453	1.412	0.310
									
12 months	Cognitive	11.760	2.620	10.000	3.000	1.7600	0.774	2.746	0.001*
	FM^2^	9.900	2.430	10.000	3.000	-0.1000	-1.067	0.867	0.838
	GM^3^	8.980	2.190	10.000	3.000	-1.0200	-1.964	-0.076	0.034*
									
24 months	Cognitive	9.100	1.520	10.000	3.000	-0.9000	-1.792	-0.008	0.048*
	FM^2^	10.160	1.350	10.000	3.000	0.1600	-0.721	1.041	0.720
	GM^3^	9.980	1.100	10.000	3.000	-0.0200	-0.887	0.847	0.964

^1^n refers to the sample size in each age group.

^2^SD refers to standard deviation.

^3^FM refers to fine motor subtest.

^4^GM refers to gross motor subtest.

*indicates significance at 0.05 level.

Male and female children performed equally well on the Bayley scales; there was also no difference in performance between children with and without siblings (p >0.05).

The test-retest interval ranged from 5 to 17 days, with a mean test-retest interval of 12.6 days. The intra class correlation (1, 1) coefficients for the cognitive, fine-motor and gross-motor scales were 0.897, 0.914 and 0.905, respectively.

## Discussion

The objective of this study was to assess the appropriateness of using the Bayley III among Sri Lankan children by comparing the cognitive and motor scores of Sri Lankan children aged 6, 12 and 24 months with that of US children.

There were some differences in the performance of Sri Lankan and US children on the cognitive and motor scales of the Bayley III. While there were no differences between Sri Lankan and US children at six months in either the cognitive or the motor domains, the cognitive scores of 12 month old SL children were significantly higher than their US counterparts (even after correcting for multiple hypotheses testing using the Bonferoni correction), but their gross motor scores were lower. At 24 months, Sri Lankan children had lower cognitive subtest scores than US children, but there were no differences in the motor scores. There were no differences in any of the subtests at any of the ages between male and female children.

The differences between Sri Lankan and US children in their gross motor scores at 12 months may be partially explained by differences in child rearing practices. It was observed that during the testing procedure that the mothers of 12 month olds did not encourage their children’s attempts to stand and walk, even with support, for fear of them falling. Sri Lankan parents, particularly those from rural areas, are reported to encourage clinging behavior in children as this keeps the child close to them, and hence safe, and actively discourage exploratory behavior [10]. Several items of the gross motor scale of Bayley III require children standing up with support, standing alone, etc. which may be the reason for SL children performing poorly on these items at this age. There were no differences between Sri Lankan and US children in the motor domain at 24 months when children had gained more control of their movements and mothers may have been less fearful.

A previous study among Asian children had reported that first birth order and female gender was associated with a child’s mental and motor scores on Bayley II [4]. In this study, there were no significant differences in the cognitive or motor scores between male and female children, or between children who had siblings and who did not have siblings. Socio-demographic factors such as father’s occupation and maternal education level were shown in a previous study to be associated with infant’s mental development [4]. The small sample size in this study did not permit meaningful comparisons between different groups; future studies should be designed to compare the performance of children from different socio-economic groups to identify predictors of infant development in Sri Lanka.

It has been observed that the length of the test can affect the performance of children, particularly at older ages. The recommended time for administration of Bayley III to children 13 months or older is 90 minutes. In this study, even though only three out of the seven subscales of the Bayley III were administered, in some instances, with 24 month olds, the test took about 90 minutes to administer. Some children got bored and lost attention or became restless and non-compliant. In order to maintain children’s interest and obtain their optimal performance, test administrators would need to develop strategies to give children breaks in between the administration of the different subscales without unduly lengthening the total administration time. It is important for test administrators to have a firm understanding and experience in testing young children [8]. Test familiarity is a key factor that results in a longer or shorter testing time. If this test is to be used in a clinical setting, test administrators, whether they are psychologists or physicians, need to be rigorously trained in the use of the test. Considering the length of the test, and the training required to administer and interpret the scores, it is not feasible for general physicians to administer this test routinely in their clinical practice.

Although there are no established standards for minimum acceptable levels of different statistics of reliability and agreement, the intra class correlation coefficients exceeded the recommended minimum value of 0.75 [11] for each of the subtests tested, indicating the test-retest stability of the Bayley III in the Sri Lankan setting.

While this study has provided important preliminary information about Sri Lankan children’s cognitive and motor development compared to US children, there are clear limitations in generalizing the findings to all Sri Lankan children. The sample was limited to a small geographic area in Sri Lanka; even though children from different ethnic and socio-economic groups were included, all the different subgroups in Sri Lanka may not have been represented. We also did not adjust for multiple hypothesis testing.

## Conclusions

There were some differences between the performance of SL and US children on the motor and cognitive scales of the Bayley III, but the differences showed no consistent age-related pattern. It can be concluded that it is feasible to use the Bayley III to assess neurodevelopment in SL children. We recommend that the Bayley III be validated for different age bands, using a larger sample of children from different geographic regions and ethnic backgrounds in Sri Lanka for it to be used as a diagnostic tool in Sri Lanka.

## Competing interests

The authors declare that they have no competing interests.

## Authors’ contributions

PG conceived and designed the study, carried out assessments and drafted the manuscript. CL and NW carried out assessments on children. AP and ARW were part of the team that conceived the study, assisted in data analysis and helped in writing and editing the manuscript. CP trained researchers, conceived the study and assisted in drafting the manuscript. NS was responsible for conceiving the study, data analysis and writing the manuscript. All authors read and approved the final manuscript.
